# Efficacy, Safety, and Economics of Innovative Medicines: The Role of Multi-Criteria Decision Analysis and Managed Entry Agreements in Practice and Policy

**DOI:** 10.3389/fmedt.2021.629750

**Published:** 2021-04-28

**Authors:** Tanja Fens, Eugène P. van Puijenbroek, Maarten J. Postma

**Affiliations:** ^1^Department of Health Sciences, University Medical Center Groningen (UMCG), University of Groningen, Groningen, Netherlands; ^2^Institute of Science in Healthy Aging and healthcaRE (SHARE), University Medical Center Groningen (UMCG), University of Groningen, Groningen, Netherlands; ^3^Department of PharmacoTherapy, -Epidemiology and -Economics, Groningen Research Institute of Pharmacy, School of Science and Engineering, University of Groningen, Groningen, Netherlands; ^4^Netherlands Pharmacovigilance Centre Lareb, 's-Hertogenbosch, Netherlands; ^5^Department of Economics, Econometrics and Finance, Faculty of Economics and Business, University of Groningen, Groningen, Netherlands; ^6^Department of Pharmacology and Therapy, Faculty of Medicine, Universitas Airlangga, Surabaya, Indonesia; ^7^Center of Excellence in Higher Education for Pharmaceutical Care Innovation, Universitas Padjadjaran, Bandung, Indonesia

**Keywords:** health technology assessment, multi-criteria decision analysis, managed entry agreements, monitoring systems, innovative medicines, regulatory policy, health-economics

## Abstract

Through the years, solutions for accelerated access to innovative treatments are implemented in models of regulatory approvals, yet with limited data. Besides efficacy data, providing adequate safety data is key to transferring conditional marketing authorization to final marketing authorization. However, this remains a challenge because of the restricted availability and transferability of such data. Within this study, we set up a challenge to analyze the answers of two questions. First, from regulatory bodies' point of view, we bring the question of whether multi-criteria decision analysis (MCDA) is an adequate tool for further improvement of health technology assessment (HTA) of innovative medicines. Second, we ask if managed entry agreements (MEAs) pose solutions for facilitating the access to innovative medicines and further strengthening the evidence base concerning efficacy and effectiveness, as well as safety. Elaborating on such challenges brought us to conclude that increasing the attention to safety in MCDAs and MEAs will increase the trust of the authorities and improve the access for the manufacturers and the early availability of safe and effective medicines for the patients.

## Introduction

Is there a perfect formula to balance pharmaceutical innovation and affordability? While the number of innovative medicines is increasing, health-care expenditure tends to go down. Currently, targeted oncological and orphan medications are the biggest challenges, testing the affordability and accessibility by different health systems. In the last decades, cost-effectiveness has become a core element health technology assessment (HTA). Ideally the cost-effectiveness should be leveled with budget impact, but obviously this is not always the case. Seen from regulatory bodies' point of view, we ask ourselves whether multi-criteria decision analysis (MCDA) is an adequate tool for further improvement of HTA of innovative medicines and if managed entry agreements (MEAs) pose solutions for facilitating the access to innovative medicines and further strengthening the evidence base concerning efficacy and effectiveness, as well as safety.

## Regulatory Efforts for Early Medicine Access

Medicines' pathways consist of development, evaluation, authorization, access, reimbursement, and adequate safety monitoring. These processes involve manufacturers/pharma-industry; international and national regulatory bodies issuing marketing authorizations (MAs); and HTA bodies that are regulating access, pricing, and reimbursement of medicines, as well as health professionals and patients. All of them are representing stakeholders that can benefit from an optimized value-based health care. Yet all have their own challenges. Further in this section, we present the perspective of the regulatory bodies and their interaction with the HTA bodies, and we trigger the interest of the patients' and manufacturers' role in the regulatory processes.

In the past years, we can notice a trend of changing models of regulatory approvals with solutions for accelerated access to innovative treatments with yet limited evidence on efficiency and safety in particular. In this set of rules, the confirmatory clinical trials are not necessarily yet part of the pre-authorization within conditional MAs. Subsequently, they can turn into a full MA after confirming the efficiency and safety in real-live observations. To facilitate these processes, regulatory authorities made adequate steps such as centralized procedure (Europe), authorization under exceptional circumstances (Europe), shorter accelerated access evaluation period (Europe, the UK, and Japan) (1–4), fast-track process (the USA), complementary regulatory process (Canada) (5, 6), or priority reviews and rapid approvals (China) (4).

Once the MA is issued, cost-effectiveness and budget impact need to be shown to justify the reimbursement of the medicine. As of that point, HTA bodies take the leading role in addressing both the pricing and the reimbursement. To facilitate this process, the European Medicines Agency (EMA) and the European Network for Health Technology Assessment (EUnetHTA) have recently allowed simultaneous advising to the manufacturers so that integrated data sets can be used for the purposes of all these bodies. Patients' opinions are included in these processes, e.g., in the early dialog of medical development, scientific advice, protocol assistance, parallel advice with HTA bodies, the initial evaluation for authorization, the development of risk minimization measures, setting up additional requirements for follow-up studies on safety, and continued monitoring of its efficacy and safety (1, 7). In the subsequent safety monitoring, patients' experiences are obviously core (7). Nowadays, registries are often used for benefit–risk monitoring [as described in risk management plans (RMPs)] of innovative medicines. A recent study has shown that such monitoring is compulsory for 9% of the centrally registered medicines, 66% of the “exceptional circumstances' registrations,” and 12% of the products with a conditional MA in Europe (8). Safety is the main focus in the majority (71%) of the aforementioned registries. While the product-oriented registries are preferred by the industry, regulatory bodies find the use of disease registries more convenient (8). The type and number of registries among various countries are given in Table 1. Furthermore, the European Network of Centers for Pharmacoepidemiology and Pharmacovigilance (ENCePP) supports the maintenance and monitoring studies for the benefit–risk balance (25). It should be emphasized that patient monitoring obtained *via* registries is open-ended and serves for continuous observations, unlike the monitoring studies that are based on research goals and have limited observational time frame (25, 26). Each of these data sources brings valuable safety information for the observed medicine or disease. Safety has an important role throughout medicines' life cycle. Therefore, filling in the gap identified in the pre-submission phase by introducing better safety setups in the early dialogs for access to innovative medicines can be beneficial for both the industry and the authorities (8, 9, 27).

**Table 1:** Some characteristics of the health-economic pathways among countries.

**Country**	**HTA focus and role in reimbursement**	**MCDA implementation elements and level**	**MEA use**	**Registries for monitoring patients**
Netherlands	HTA and BIA are used with focus on necessity, effectiveness, safety, and efficiency.	System accounts for equity and severity in differential thresholds using proportional shortfall. Horizon scanning is also used.	Often applied, mostly financial based, and few outcomes-based MEAs. Focuses primarily on evidence generation.	Disease-specific registries exist, e.g., 5 active registries up to 2013 for medicines subject to CMA.
Norway	HTA is used as a final tool for reimbursement.	System accounts for severity (severity of illness by absolute shortfall) in differential thresholds. Horizon scanning is also used.	There is a legal framework for MEA, and each medicine is under a separate contract.	Disease-specific registries exist, e.g., 2 active registries up to 2013 for medicines subject to CMA.
France	HTA is used supplementary to underpin the reimbursement decision.	System recognizes the need for diseases with high severity and shows elements of value-based pricing. Horizon scanning is also used.	Financial and primarily evidence generation MEAs are used. Potentially less transparent than in some other countries.	Disease-specific registries exist, e.g., 12 active registries up to 2013 for medicines subject to CMA.
Spain	HTA is well-established based on assessment of safety, efficacy, and efficiency.	System recognizes the need for severity.	Financial and financial linked to optimizing utilization MEAs are identified.	Disease-specific registries exist, e.g., 6 active registries up to 2013 for medicines subject to CMA.
Germany	System accounts for clinical outcomes including additional benefits over the comparator dominantly, with no role of HTA.	System recognizes the need for severity. It also has elements of value-based pricing.	Financial and financial linked to optimizing utilization MEA are identified.	Disease-specific registries exist, e.g., 10 active registries up to 2013 for medicines subject to CMA.
Sweden	HTA serves as final tool for reimbursement considering cost-effectiveness and the clinical evidences.	System recognizes target population and comparator and recognizes the need for severity. It has elements of value-based pricing. Horizon scanning is also used.	There is primarily evidence generation (coverage with evidence development).	Disease-specific registries exist, e.g., 6 active registries up to 2013 for medicines subject to CMA.
Italy	HTA is used supplementary to the reimbursement decision.	System accounts for value-based pricing. Horizon scanning is also used.	Existence of outcome-based and financial MEA. Accounted for high-prized oncology products.	There are available comprehensive systems for data collection on pharmaceutical use in clinical practice, which facilitates post-marketing surveillance. Disease-specific registries also exist, e.g., 10 active registries up to 2013 for medicines subject to CMA.
Czech Republic	HTA is used supplementary to the reimbursement decision.	System recognizes potential MCDA criteria for consideration: efficacy/effectiveness, safety, budget impact, disease severity, cost-effectiveness, and unmet needs.	Financial (discounts, price–volume agreements, pay-back) and health outcome-based (payment by result) MEA.	Disease-specific registries exist, e.g., 1 active registry up to 2013 for medicines subject to CMA.
Poland	Several types of HTA submissions are used: clinical assessments, economic analyses, BIA, and rationalization analyses. The transparency improves through the years.	System has elements of value-based pricing.	Financial (discounts, price–volume agreements) and health outcome-based (bundle and other agreements, payment by result) MEA.	Disease-specific registries exist, e.g., 1 active registry up to 2013 for medicines subject to CMA.
Ireland	Compulsory rapid reviews are required for all new medicines after a licensing decision, and based on those, HTA is further required or not required.	System has elements of value-based pricing.	There is primarily evidence generation, and there are patients' access schemas for particular products.	Disease-specific registries exist, e.g., 4 active registries up to 2013 for medicines subject to CMA.
England	HTA is used as final tool for reimbursement. CEA and cost/QALY are dominating.	System accounts for background disease info, population, comparators, evidence based, health outcome measures, equity, severity of illness (end of life care), and rarity. Horizon scanning is also used.	There are transparent high-prized oncology products, and patients' access schemas for particular products exist. Dominantly financial MEA are used, but outcome-based MEA is also performed.	Disease-specific registries exist, e.g., 9 active registries up to 2013 for medicines subject to CMA.
Canada	HTA is used accounting for cost and cost-effectiveness measures, clinical efficacy, and clinical safety.	System accounts for target population, comparators, outcome measure, equity. Horizon scanning is also used.	Reimbursement/coverage agreements are used.	Disease-specific registries exist.
Belgium	Several types of HTA submissions are used: Cost-effectiveness or cost utility, budget impact and clinical effectiveness.	System accounts for target population, comparator, ethical issues, equity, impact on the health system.	Transparent policy using financial MEA (e.g., budget cap).	Disease-specific registries exist, e.g., 4 active registries up to 2013 for medicines subject to CMA.

*HTA, health technology assessment; MCDA, multi-criteria decision analysis; MEA, managed entry agreement; BIA, budget impact analysis; CMA, centralized marketing authorization; CEA, cost-effectiveness analysis; QALY, quality-adjusted life-year*.

*NB. The table information is authors' extraction data based on the following references: (8–24). Given the dynamic nature of health polices among countries, presented data may differ from the exact current health-care setting and policy in particular countries*.

While regulatory bodies are making efforts to get the processes to work, manufacturers are more and more pushed to show excellence in reporting and fulfilling the payor-specific needs while demonstrating the real-world value of the newly introduced medicines. The challenge is to optimally integrate this with HTA processes, organization, and outcomes.

## Health Technology Assessment in Practice and Policy

HTA uses a rather broad spectrum of elements to inform the decision makers. The information however is predominantly based on the economic analyses, in particular cost-effectiveness analyses (CEAs) and cost utility analyses if quality-adjusted life-year (QALY) is the measurement for consequence. CEAs for new treatments are used to leverage the (relative) effectiveness and safety over the standard/common treatments and costs, as well as to demonstrate authorities if the new medicine is investment worthy. Such measurement of allocative efficiency usually shows extra costs/expenses but also more effects than the standard treatment; nevertheless, ideally of course, it should show less expenses combined with higher effectiveness (28). This challenge becomes even bigger when discussing innovative medical solutions. At least two questions are arising: how can manufacturers maximize the value of their innovative medicines and do the criteria used in conventional HTA sufficiently capture all aspects of value (29–31)?

Utilization of conventional HTA is widely accepted; however, certain countries are recently deciding to opt in toward a more MCDA-attributed HTA. Conventional HTA traditionally has one or just a few aspects dominating. The incremental cost-effectiveness ratio (ICER), usually representing cost per QALY, is taken as a common measuring value. Yet it seems not always to be sufficient because it does not capture all aspects of value, notably with relevant aspects of safety often being missed (29, 30). In that context, MCDA may be better suited for evaluating the value of health-care interventions. Moreover, MCDA is already used to show the consistency benefit–risk balance in the benefit–risk assessments (32). In the pathway of converting conventional HTA to MCDA, several modifiers could be considered on the standing conventions: proportional shortfall, differential willingness to pay (WTP) by burden, severity, equity, rarity, end of life, fair innings, and evidence on safety (10, 11, 33).

The ICER could be integrated together with these modifiers to better reflect the value. For example, the proportional shortfall may pose a solution in regard to QALY equity rather than using the standard incremental approach, where QALYs are attributed with equal value for any disease burden (11, 34). Furthermore, the threshold that distinguishes between “passed” and “failed” in cost-effectiveness seems arbitrary and potentially not justifying its major sole role that it has in some countries. Moreover, thresholds are variable between countries, both numerically and structurally, as some countries use fixed and others differential thresholds. In the Netherlands and Norway, the cost-effectiveness threshold is proportionally dependent on the burden of disease, and differential thresholds apply. Severity is sometimes related to the differential thresholds, but not necessarily (11, 12). Incorporating equity in the ICER calculation would insinuate adding societal preferences to cost-effectiveness. Moreover, assigning equity weighting to the QALY will allow more perspicuous allocative decision making (11, 35, 36). Rarity plays an important role in defining the value of the orphan medicines (33, 37). End of life, as a criterion for value, is usually attributed to interventions accompanied with the highest threshold, like those applied in England (38). Evidence on safety within a MCDA provides parameter estimation but surely represents a challenge to reflect societal implications. Those can be addressed by new approaches such as safety by design into MCDA (39). As application of one modifier may not grasp all details of the QALYs, a multi-value approach is worth considering (40). Notably, full integration of safety aspects, inclusive mental as well as physical aspects, in the QALY remains a challenge.

Several frameworks and initiatives tried to structure the MCDA criteria. The Evidence and Value Impact on DEcisionMaking (EVIDEM) framework is often seen to be used as a MCDA analytical tool, accounting for disease severity, population, urgent needs, comparative effectiveness, comparative safety/tolerability, comparative health, comparative costs, benefit, quality of evidence, clinical practice guidelines, and value evidence (41, 42). The BEACON framework stands for burden/target population, environment, affordability/value, comparator, outcomes, and number of studies/quality of evidence (15). Disease-oriented frameworks can also be found. The European Society for Medical Oncology (ESMO) uses the ESMO-Magnitude of Clinical Benefit Scale (MCBS) tool to quantify value in cancer care (43). The same focus remains in The American Society of Clinical Oncology (ASCO) and the National Comprehensive Cancer Network (NCCN) (44, 45).

Some suggest a value-matrix frame to quantify the measurements with MCDA and serve as a quality assessment tool in combining HTA and MCDA (46, 47). Despite the effort for structuring MCDA, the methodologies applied remain heterogeneous. Some examples include discrete choice experiment (DCE), Potentially all Pairwise Ranking of all possible alternatives (PAPRIKA), analytic hierarchy process (AHP), Measuring Attractiveness by a Categorical Based Evaluation TecHnique (MACBETH), Simple Attribute Rating Technique (SMART), direct weighing, weighted benefit scores (WBSs), and Elimination and Choice Expressing Reality (ELECTRE) (10, 48, 49). While some suggest that the commonly used technique for value in MCDA quantification is weighted sum approach (50), others emphasize the AHP and the multi-attribute utility theory (MAUT) as the most common (51). New approaches combining MCDA and WTP have also been explored (51). In one approach, the WTP is closely related to safety outcomes of the new interventions (52). Yet the aforementioned frameworks do not consistently use all the value of safety while preforming MCDA.

In the case of one solution fits all, the health-care systems would remain homogenous. However, HTA bodies vary through the sets of national priorities. Every country has their own reimbursement and pricing policy (relevant characteristics of HTA per country are given in Table 1). An important challenge concerns the integration of national HTAs into multinational collaborations. Beside the existence of the EUnetHTA, which aims to unify the HTA methodologies, HTA is still used for different purposes. For example, the HTA in France, Italy, and Czech Republic is used as supplementary advice for reimbursement or price decisions of the health authorities; while in Norway, Sweden, and England, it is used as a tool for final reimbursement or rejection of funding (13). Other examples from Europe, e.g., Germany, show transparency in prices discounting and mainly focuses on clinical outcomes. Unlike Germany, in the UK, the focus is on CEA, with cost/QALY dominating, but potentially less transparent. In Sweden, pricing processes are transparent, clinical evidence is required, and CEA is considered (9). The Dutch system can be conceived as approaching MCDA. Even though not completely transparent, societal perspective is applied together with the care about the medical need, cost-effectiveness, quality of life, budget impact, efficacy, effectiveness, and safety as well as severity as a separate criterion (11). More details about the HTA focus and role in reimbursement, as well as some implementation elements and level of MCDA, are given in Table 1.

Furthermore, the budget impact analysis (BIA) can be complemented by horizon scanning used to prioritize the fund allocation. This tool is used in Italy, France, Sweden, Norway, England, the Netherlands, and Canada. Yet establishment and maintenance of such tool pose concerns regarding ethics and data exchange and is a time-consuming activity (14, 53). A recent study addressing the problem of the rare disease non-alcoholic steatohepatitis showed that MCDA is promising in supporting early HTA, illustrating high consistency in results across England, France, and Germany (54).

## Managed Entry Agreements in Practice and Policy

While the World Health Organization (WHO) is actively working on providing equal access to essential medicines, innovative medicines remain in the hands of the manufacturers and patent holder, so it remains a challenge to arrange access at affordable prices and circumstances for society (55). Conceived high medicine prices are reported to remain the main barrier to be overcome in health-care systems (56). Furthermore, the health-care expenditure is related with gross domestic product (GDP) representing the wealth of one country. Consequently, developed countries are more likely to have higher health budget and therefore better chance for access to innovative medicines than the developing ones (57).

**Figure 1:**
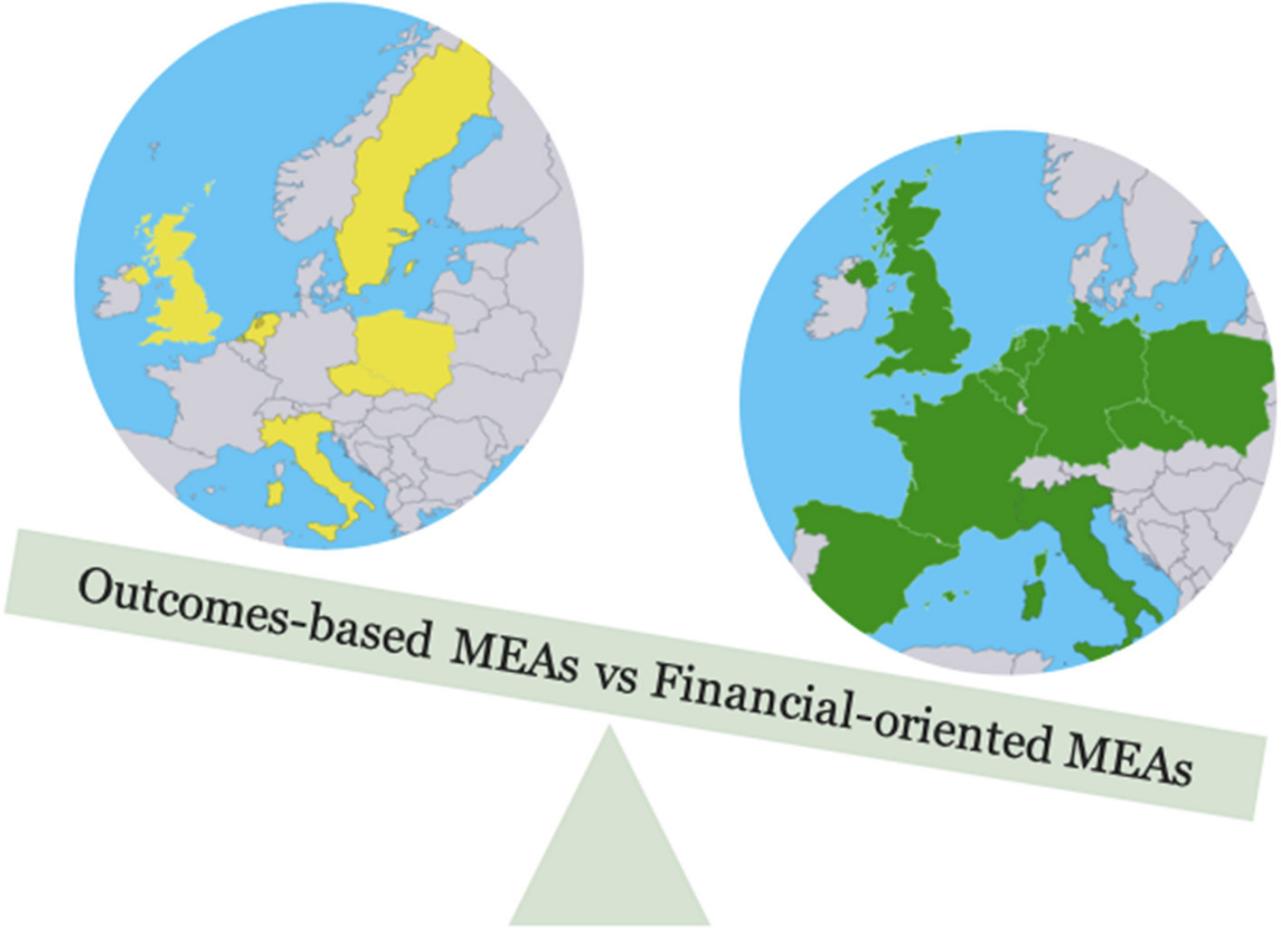
Type of managed entry agreements across countries. MEA, managed entry agreement.

MEAs were introduced as a tool for overcoming these accessibility barriers but also to mitigate the uncertainty attributed to economic evaluations and BIAs accounting for the real-world evidence (58). Often, MEAs represent an integrated effort by the manufacturers on the one hand and the health authorities, Ministries of Health—potentially with other ministries, like the Ministry of Economics or Finance—on the other hand (27). MEAs are often differentiated into financial-oriented and outcome-based ones. Illustrative examples of both are a budget cap and no-cure/no-pay, respectively (59). While the benefits of MEA are obvious, there are remaining challenges to be addressed. The global trend of MEA reflects dominant utilization of the financial-based ones, like in England, Portugal, Lithuania, Belgium, and Cyprus. Outcome-based MEA are seen in the Netherlands, Sweden, and Czech Republic (58, 60). Further details over MEA country specifics are given in Figure 1 and Table 1. To be noted, MEA is part of a dynamic field, and countries' preferences change easily; for example, the Netherlands switched from outcome-based MEA to price negotiations (59).

Nevertheless, a recent study brings to attention that the increased availability of innovative medicines is in line with the increased number of MEA, giving real-world evidence from around the globe (61).

In the regulatory pathway, outcome-based MEA was not commonly used for the products with conditional MAs, yet monitoring is more likely to accompany outcome-based MEAs than the financial-based ones (8, 27). Furthermore, as manufacturers provide additional data to support their conditional MA, authorities seem not always to have all the information needed. Divergence appears in the requirements of HTA and regulatory bodies when further proof of efficiency and safety needs to be provided. While HTA bodies in one adaptive pathway require observational study outcomes, the regulatory authorities stick to randomized controlled trial (RCT) evidence for the Phase III. Unlike the payors and HTA bodies, manufacturers are keen toward value-based MEA, posing a good solution for market positioning of their products.

## Actionable Recommendations While Using Multi-Criteria Decision Analysis and Managed Entry Agreements

Among the many advantages complementing the HTA with MCDA, potential limitations may appear (15, 50, 62). To overcome such posed challenges, concrete actions are required. Therefore, we recommend:

- setting up criteria in which medicines are eligible for MCDA,
- specifying the selection and weighting criteria,
- choosing the “right model” for the investigated intervention,
- setting up guidelines and methods for unified outcome interpretation,
- presenting a clear definition of the value perspective and accounting safety in this context,
- budget consideration, and
- handling uncertainty.

Having this addressed will bring us closer to the answer if MCDA becomes the new standard for HTA appraisal.

When the access of innovative medicines in relation to MEAs is concerned, there is room for improvement in diverse national settings' functionalities and implementation. In this matter, many initiatives are undertaken to facilitate these processes, such as Shaping European Early Dialogues (SEED), aiming to support the mutual interests of HTA bodies and manufacturers through overcoming the national obstacles regarding data from the initial phases of clinical development (15). Furthermore, the International Coalition of Medicines Regulatory Authorities (ICMRA) already joined forces with all regulatory authorities toward facilitating accelerated procedures and equitable access to global clinical trials data regarding COVID-19. Moreover, they emphasize the importance of further monitoring for reflecting the real-life situation (63). This current setup might serve as an example for establishing better monitoring platforms for innovative medicines as subjects of MEAs. Bearing in mind the importance of real-life observations, effectiveness and safety reporting are pivotal data to be exchanged between the health authorities. Therefore, further development of activities such as EMA Registries Initiative is important and welcome (8).

MEA-related recommendations for better practices:

- establishing mutual/shared monitoring platforms for innovative medicines that would allow data exchange across the countries and save time and resources and
- support of collaborating initiatives for safety monitoring registries.

## Conclusion

The access to innovative medicines can be facilitated through several go-to-market mechanisms. It might be beneficial to use adaptive pathways where RA and HTA provide early advice accounting safety. In addition, considering migration toward MCDA will highlight more aspects of value, account for safety, and give manufacturers “a joker” for a next step further in the access. More research is needed to prioritize the value elements, position the safety, and establish a common MCDA structure. Additionally, MEAs appear to pose a good solution for access of innovative medicines. However, further work should be done on solutions to overcome the obstacles around monitoring platforms in regard to patient safety in particular, sharing blinded data among countries to prevent double work and fasten submission processes. Increasing the attention to safety in MCDAs and MEAs will increase the trust of the authorities and improve the access for the manufacturers and the early availability safe and effective medicines for the patients.

## Author Contributions

The study was initially conceptualized by TF and MJP. TF wrote the first draft and extracted the data used in the table, which further were reviewed, adjusted, and edited together with EPvP and MJP. All authors have read and agreed to the published version of the manuscript.

## Conflict of Interest

The authors declare that the research was conducted in the absence of any commercial or financial relationships that could be construed as a potential conflict of interest.
